# Preoperative Templating Prior to Total Hip Arthroplasty: A Closed-Loop Audit

**DOI:** 10.7759/cureus.77083

**Published:** 2025-01-07

**Authors:** Conor J Kilkenny, Conor O'Driscoll, Samher Jassim, Martin S Davey, Niall P McGoldrick, John F Quinlan

**Affiliations:** 1 Trauma and Orthopaedics, Tallaght University Hospital, Dublin, IRL

**Keywords:** arthroplasty, clincal audit, implant selection, pre-operative planning, preoperative templating

## Abstract

Background: Preoperative templating is an essential process in total hip arthroplasty (THA), aiding surgical planning and implant selection to optimize patient outcomes. Despite its significance, templating is inconsistently practiced, influenced by factors such as surgeon experience and institutional protocols. In light of increasing THA rates, this study aimed to assess adherence to templating guidelines within our institution and evaluate the impact of a new protocol on compliance.

Methods: We conducted a closed-loop audit with an initial review of 50 THA cases, assessing the frequency and timing of preoperative templating. Following the first audit cycle, a departmental protocol was introduced, mandating templating radiographs 7-180 days before surgery, supported by a departmental rota. In the subsequent audit cycle, another 50 patients were reviewed. Templating was performed using TraumaCad software (Orthocrat Ltd, Petach Tikva, Israel) on anteroposterior pelvic radiographs with a calibration sphere. Statistical analysis was conducted using Welch's unpaired t-test. Leg length changes were evaluated in all patients based on their two most recent radiographs during the second audit cycle to determine if leg lengths had changed over time.

Results: In the pre-intervention cycle, only 20% of patients had templating radiographs within the recommended timeframe, with an average of 140 days between the most recent radiograph and surgery. Post-intervention, compliance improved significantly, with 70% of patients receiving templating radiographs within the specified period (p < 0.0001), and the average time between the most recent radiograph and surgery reduced to 33 days. A mean leg length change of 2.26 mm was observed, with changes ranging from 0 to 17 mm between radiographs. The mean interval between radiographs was 386 days.

Conclusion: This closed-loop audit found that implementing booking preoperative templating radiographs prior to THA and integrating them as part of the preoperative assessment process had a significant impact on the number of patients having appropriate imaging performed preoperatively. Simple interventions, such as the introduction of a roster, helped ensure compliance in the booking of departmental radiographs. This study has shown how patient parameters can change over time and emphasizes the importance of ensuring up-to-date imaging to allow accurate surgical planning.

## Introduction

Preoperative templating is an essential component of total hip arthroplasty (THA), aiding in surgical planning. Preoperative templating helps guide the selection of implants and allows surgeons to anticipate potential case-specific difficulties, which can be addressed and accounted for preoperatively, aiming to optimize patient outcomes [1-3]. However, despite its importance, there appear to be inconsistent templating practices across institutions. The number of THA procedures performed continues to grow [4], making it crucial to implement standardized templating protocols to enhance surgical accuracy.

Litigation is common following THA, particularly if any surgeon- or technique-specific errors are found [5]. Templating provides surgeons with a detailed preoperative plan, aiding in the accurate sizing and positioning of prosthetic components to restore normal hip biomechanics, such as limb length and femoral offset [6]. Appropriate planning can help minimize complications and improve the accurate restoration of hip biomechanics, thereby enhancing surgical outcomes and patient function [7]. However, despite its advantages, there is variability in its application across institutions and among surgeons. Factors such as surgeon experience, institutional protocols, and the availability of templating software likely contribute to this inconsistency. There is limited reporting on the application of preoperative templating across institutions.

To address this, we conducted a closed-loop audit within our institution to assess the frequency of preoperative templating in patients undergoing hip arthroplasty. According to guidelines by the New Zealand Orthopaedic Association and the South African Arthroplasty Society, preoperative templating should be performed on all patients undergoing THA [8,9]. Guidelines on the preoperative workup of patients prior to THA, known as the "Getting It Right First Time" (GIRFT) guidelines from the UK, recommend that all radiographs be performed within six months prior to referral to orthopedics [10].

The aim of this study was to determine the extent to which templating is performed within our institution and to identify whether the implementation of a departmental strategy would improve adherence to the above guidelines. Our goal was for all patients to receive preoperative templating within 7-180 days as per the guidelines. This timeframe would allow appropriate preparation if specific implants or equipment were required before the procedure while ensuring radiographs were performed at an appropriate time point. We hypothesized that our department's current preoperative templating protocols would fall below current guidelines.

## Materials and methods

Following approval granted on July 15, 2024, to perform an audit by the Tallaght University Hospital Clinical Audit Board (Audit Number 4558, 2024), a retrospective review was conducted of 50 patients who underwent THA in the elective setting in our institution over a three-month period. Their electronic medical records were accessed by two independent reviewers (CJK and COD) to analyze the preoperative templating standards within the department. Thereafter, data verification was performed by a third reviewer (SJ). As no clinical data were being collected or analyzed, and as this was an audit of standards, no informed consent was ethically required from patients prior to the commencement of the audit process.

Data recorded included the following: (1) patient board number at our institution, (2) name of the proposed procedure, (3) date of surgery, (4) date of preoperative radiograph, (5) number of days between radiograph and surgery, and (6) whether templating was performed between 7 and 180 days.

After preliminary analysis by the principal investigator (CJK) and senior investigators (NPMcG and JFQ), it was decided that a further audit cycle would be required to evaluate the standards. The audit committee proposed that a minimum of 50 additional patients be included to assess for a sufficiently powered quality improvement. A new departmental protocol was implemented, requiring all patients to undergo preoperative assessment prior to THA, which included preoperative templating radiographs. All patients included in this audit were a consecutive group during the study period, representing cases from all seven arthroplasty surgeons within the department. Non-Consultant House Doctors (NCHDs) in the department were provided education regarding the appropriate radiographs to obtain preoperatively. Radiographic evaluation was conducted using standing anteroposterior (AP) hip radiographs. All AP radiographs were taken with a film focus distance of 1.15 meters, following a standardized protocol in which both feet were internally rotated by 10°-15°. The radiographs were centered to ensure the symphysis pubis was positioned directly below the coccyx, with both obturator foramina appearing symmetrical. To ensure compliance, a departmental rota was introduced, assigning NCHDs responsibility for ordering the appropriate radiographs. A further audit cycle was conducted prospectively from August 1, 2024, to November 5, 2024. All preoperative templating was performed by the operative surgeon or surgical trainees on the anteroposterior pelvic radiograph (with a 25.4 mm calibration sphere) using TraumaCad software (Orthocrat Ltd, Petach Tikva, Israel) and an assumed 118% magnification correction as previously established [11]. 

Following the second audit cycle (i.e., closing the loop), statistical analysis was carried out using the online tool GraphPad Calculator (https://www.graphpad.com/quickcalcs/ttest2/). A Welch’s unpaired t-test was performed to analyze the differences in the rate of preoperative templating performed between 7 and 180 days during the initial audit cycle and the post-intervention cycle. A p-value of less than 0.05 was deemed statistically significant. Power analysis was conducted using the online tool Sealed Envelope (https://www.sealedenvelope.com/power/binary-superiority/). A superiority trial was utilized with a 5% significance level, powered at 90%, and a percentage success rate of 50%.

To evaluate the importance of using up-to-date radiographs when performing preoperative templating, leg length discrepancies were recorded for all patients included in the post-audit cycle based on their most recent radiographs. A leg length discrepancy was also measured on their previous radiograph to assess changes over time. Radiological assessment of leg length was performed using an AP pelvic image by drawing a line through Köhler’s teardrop figure and aligning it parallel to the lesser trochanter.

## Results

Preintervention cycle

During the initial audit cycle, a total of 50 patients underwent THA. The number of days between the patient’s most recent radiograph and the date of surgery ranged from 0 to 788 days, with an average of 140 days. Ten out of the 50 patients (20%) had templating radiographs performed within 7 to 180 days (Table 1).

Postintervention cycle

Following the intervention, a further 50 patients underwent THA. The number of days between the patient’s most recent radiograph and the date of surgery ranged from 0 to 172 days, with an average of 33 days. Thirty-five out of the 50 patients (70%) had templating radiographs performed within 7 to 180 days. This represents a 50% improvement from the initial audit cycle (p < 0.0001) (Table 1).

Power analysis

The power analysis determined that a minimum sample size of 96 was required, with a minimum of 48 per group.

Leg length changes

Leg length changes were measured for all patients included in the post-intervention cycle. A mean leg length change of 2.26 mm was observed, with changes ranging from 0 to 17 mm between radiographs. The mean number of days between radiographs was 386 days.

**Table 1:** Templating Results

Variables	Cycle 1	Cycle 2	P-Value
Total	50	50	N/A
Average number of days between X-ray and surgery	140	33	N/A
Templating performed between 7 and 180 Days	10 (20%)	35 (70%)	<0.0001

## Discussion

The most important finding of this study is that a statistically significant change was observed in the number of preoperative templating radiographs performed within our institution in an appropriate time frame. This is particularly important given that we have shown that crucial parameters, such as leg length discrepancies, change over time. Following the integration of a new departmental protocol, a 50% increase in compliance with preoperative templating prior to THA was observed.

When performing preoperative templating, one should follow the intraoperative order of surgery, starting with the acetabulum [12]. Templating involves the identification of key anatomical landmarks. For acetabular implant positioning, anatomical landmarks such as the teardrop, acetabular roof, ilioischial line, and superolateral margin of the acetabulum should be identified [1]. Radiological leg length can be calculated on an AP pelvic radiograph by measuring a fixed point on the femur and on the pelvis. Common landmarks used include the vertex of the lesser trochanter and the inferior aspect of the acetabular teardrop on the pelvis, ischial tuberosities, or the obturator foramina [5]. Clinical examination remains crucial, as this form of templating assumes leg lengths are equal from the lesser trochanter downward [1]. Placement of the acetabular component has been described by Lewinnek et al. as being in a "safe zone" of 15 ± 10 degrees of anteversion and 40 ± 10 degrees of inclination [13]. Correct positioning and orientation are paramount to ensure stability and to prevent complications such as impingement, osteolysis, leg length discrepancy, and accelerated implant wear [14,15].

For the femoral component, key anatomical landmarks include the greater trochanter, lesser trochanter, and medullary canal [12]. Preoperative templating allows the surgeon to plan for appropriate femoral offset while maintaining correct leg length [5]. A planned neck cut can be measured from the tip of the lesser trochanter and matched intraoperatively on the femur [5]. Attention should be given to the type of femoral stem planned, particularly whether it is cementless or cemented, as different stem types have different fixation modes. For cemented stems, different femoral stem types require varying amounts of a cement mantle, which will affect the stem size for a given medullary canal [16]. For proximally coated cementless stems, good contact should be ensured between the lateral and medial endosteal cortex of the proximal femur. In contrast, extensively porous-coated stems require bone contact throughout the diaphysis for fixation [12,17].

Several methods of preoperative templating have been described in the literature [18]. All templating in this study was performed using digital templating via TraumaCad. Digital templating appears to have replaced more conventional or analog preoperative planning and is currently considered the gold standard for preoperative templating. Digital radiographs reduce radiation exposure and have fewer instances of over- and underexposure, with lower rates of inadequate radiographic images [19]. Digital templating involves the use of software to aid in preoperative planning for arthroplasty by calculating radiograph magnification and adjusting the templates to match the exact magnification [20]. Digital templating has been reported to have increased accuracy compared to conventional templating and has been recommended as routine practice prior to arthroplasty [21,22]. However, Dutka et al. found that conventional templating was more accurate than digital templating regarding acetabular cup planning and femoral stem size [23]. Recently, new 3D-based CT templating technology has emerged, which is believed to further increase accuracy in preoperative planning [24-28].

Research has shown that orthopedics is one of the specialties most at risk for litigation, with 78% of orthopedic arthroplasty surgeons in the US being involved in at least one lawsuit due to alleged medical malpractice [29-31]. The most common causes of litigation post-THA include death, LLD, periprosthetic fracture, and hip dislocation [32]. The findings of our study emphasize the importance of using up-to-date templating radiographs preoperatively, as changes in leg length occur over time and it is crucial that surgeons are aware of these discrepancies before surgery. With the majority of litigation resulting from technical errors, preoperative templating is vital as it can improve accuracy and potentially reduce such errors [5]. Plaintiffs appear to be more successful when they can prove that a complication resulted from a technical error [31,33]. Training and education for future arthroplasty surgeons should focus on meticulous surgical techniques and preoperative planning to help reduce their risk.

Limitations

While this closed-loop audit improved standards within this institution and achieved its aims, it is not without limitations. First, the audit was conducted over a relatively short period of approximately three months. The templating performed preoperatively was often carried out by surgical trainees who may not have been performing the procedure, which could limit its effectiveness. Leg length measurements were conducted using AP pelvis radiographs; further accuracy could be obtained using full-length lower limb radiographs.

## Conclusions

This closed-loop audit found that the implementation of booking preoperative templating radiographs prior to THA and integrating them into the preoperative assessment process had a significant impact on the number of patients receiving appropriate imaging preoperatively. Simple interventions, such as the introduction of a roster, helped ensure compliance in the booking of departmental radiographs. This study highlights how patient parameters can change over time and emphasizes the importance of ensuring up-to-date imaging to facilitate accurate surgical planning.
